# Gut Microbiota and Chemical-Induced Acute Liver Injury

**DOI:** 10.3389/fphys.2021.688780

**Published:** 2021-05-26

**Authors:** Tao Chen, Rui Li, Peng Chen

**Affiliations:** ^1^Department of Physiology, School of Basic Medical Sciences, Gannan Medical University, Ganzhou, China; ^2^Department of Pathophysiology, Guangdong Provincial Key Laboratory of Proteomics, School of Basic Medical Sciences, Southern Medical University, Guangzhou, China

**Keywords:** chemical, gut-liver axis, gut microbiota, intestine, acute liver injury

## Abstract

**Background:** Drug overdose or chemical exposures are the main causes of acute liver injury (ALI). Severe liver injury can develop into liver failure that is an important cause of liver-related mortality in intensive care units in most countries. Pharmacological studies have utilized a variety of comprehensive chemical induction models that recapitulate the natural pathogenesis of acute liver injury. Their mechanism is always based on redox imbalance-induced direct hepatotoxicity and massive hepatocyte cell death, which can trigger immune cell activation and recruitment to the liver. However, the pathogenesis of these models has not been fully stated. Many studies showed that gut microbiota plays a crucial role in chemical-induced liver injury. Hepatotoxicity is likely induced by imbalanced microbiota homeostasis, gut mucosal barrier damage, systemic immune activation, microbial-associated molecular patterns, and bacterial metabolites. Meanwhile, many preclinical studies have shown that supplementation with probiotics can improve chemical-induced liver injury. In this review, we highlight the pathogenesis of gut microorganisms in chemical-induced acute liver injury animal models and explore the protective mechanism of exogenous microbial supplements on acute liver injury.

## Introduction

Acute liver injury (ALI) is a common disease that seriously threatens the life and health of the patients. The main pathological manifestation is a sharp decline in liver function caused by the necrosis of a large number of hepatocytes (Lee, 2004; Bernal et al., 2010; Bunchorntavakul and Reddy, 2018). ALI caused by multiple etiologies has become a crucial public-health issue at both regional and global scales. The most common causes of ALI in developed countries are acetaminophen (APAP) induced hepatotoxicity, drug induced liver damage, autoimmunity, and viral hepatitis B, which accounts for about 70% of the cases; on the other hand, the most common causes in developing countries are viral hepatitis A, B, and E (Stravitz and Lee, 2019). Currently, liver transplantation (LT) is the only clinically essential therapy for the treatment of acute liver failure (ALF). However, the rapid progression of ALI, the scarcity of liver sources, and the high medical costs limit its application. Therefore, it is necessary to find other effective interventions for severe acute liver injury patients. Recently, there is increasing evidence points to the intimate connection between gut microbiota and liver injury. However, compared with ALI, these studies mostly focused on chronic liver diseases, such as non-alcoholic fatty liver disease (NAFLD), autoimmune hepatitis, liver cirrhosis, and hepatocellular carcinoma (Compare et al., 2012; Lee and Suk, 2020; Wei et al., 2020). In recent years, an increasing number of preventive or adjunct therapeutic measures for ALI have been proposed based on the regulation of gut microbiota (Kim et al., 2018; Jiang et al., 2021). The gut contains around 1,000 different species of bacteria, which play a crucial role in the survival of organisms. The crosstalk between gut and liver is increasingly recognized. Gut microbiota has been defined as a significant micro-ecosystem, which is symbiotic with the organism and participates in a variety of physiological and pathological processes (Sharma and Gilbert, 2018).

In this review, we focused on the current knowledge of the gut microbiota's contribution and the protective mechanism of exogenous microbial supplements on chemically induced ALI.

## Role of the Microbiota in the Immune Homeostasis of the Gut, Liver and Liver Disease

### Immune Homeostasis of the Gut and Liver

**Figure 1:**
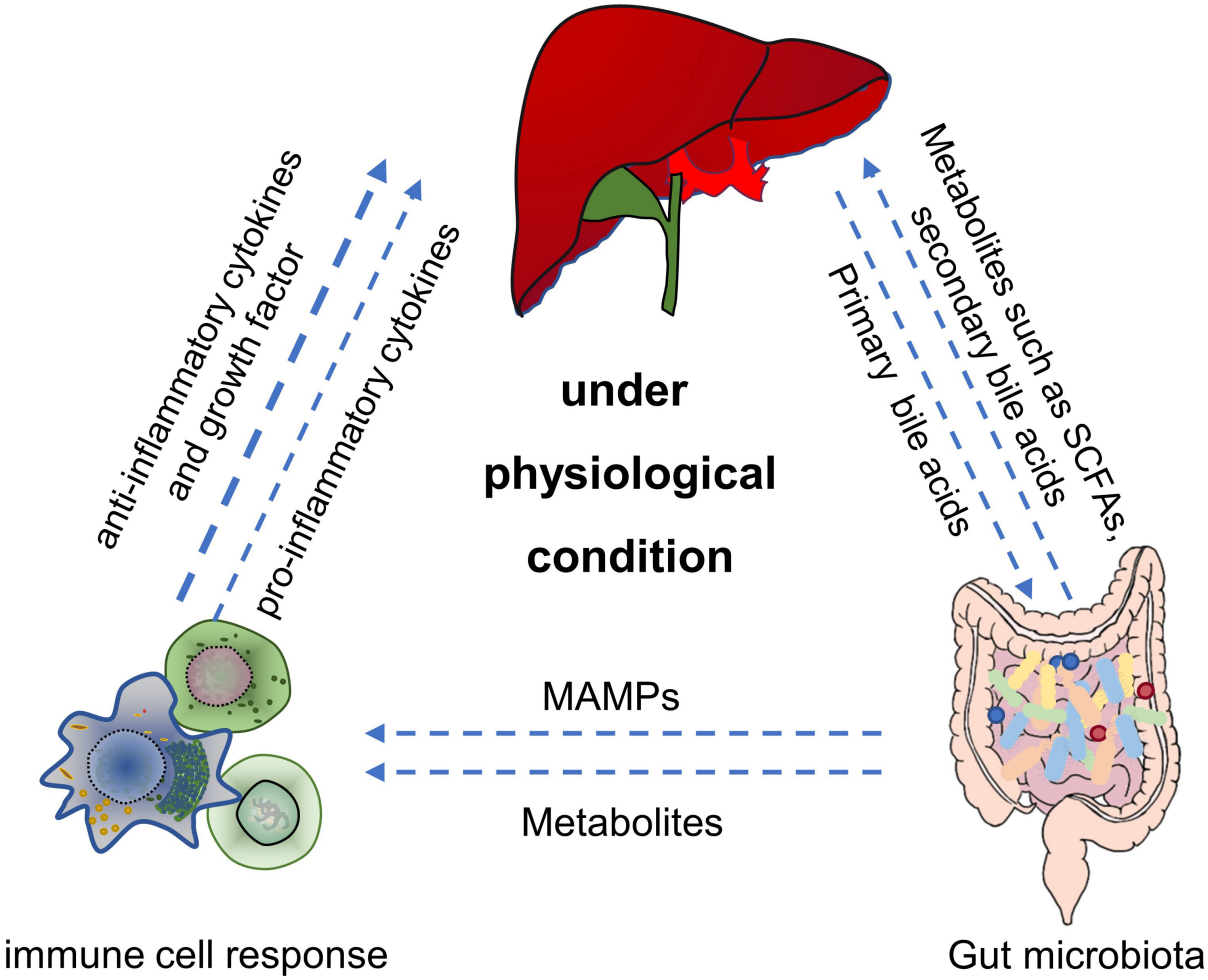
The main role of gut microbiota in gut-liver-immune axis under physiological condition. First, by utilizing the nutrients and metabolic substrates (such as primary bile acids from liver), gut microbiota can produce various bioactive metabolites (such as acetate, propionate, butyrate, secondary bile acids, and amino acids) which were absorbed through the portal vein into the liver, to regulate hepatic function. Second, gut microbiota can also directly or indirectly establish and balance the hepatic immune response through metabolites, secondary bile acids and MAMPs. SCFAs, short chain fatty acids. MAMPs, microbial-related molecular patterns. Figure was partly adapted from Smart Servier Medical Art (https://smart.servier.com/), which is licensed under a Creative Common Attribution 3.0 Generic License.

The immune system plays a critical role in maintaining the symbiotic relationship between the microbiome and the host. Intestinal mucosa-associated lymphoid tissue contains a variety of immune cells, such as antigen-presenting cells (APCs), innate lymphocytes, T cells, and B cells, which play a vital role in the host immune response (Trivedi and Adams, 2016). Gut bacteria regulate the maturation of the mucosal immune system, which in turn affects the composition of the gut microbiota (Shan et al., 2013). Studies have shown that microbes activated the immune cells to produce cytokines and initiated the host's immune response to balance the intestinal tolerance and immunity (Venkatesh et al., 2014; Rooks and Garrett, 2016). Thus, the homeostasis of the gut microbiota is essential for the maintenance of gut and liver metabolism and immune homeostasis (Figure 1). For example, intestinal symbiotic bacteria help induce a stable production of Th17 cells in the lamina propria of the small intestine and protect the intestinal mucosal barrier, and some bacteria also play an immunomodulatory role of macrophage polarization (Scott et al., 2018; Wang et al., 2020c).

Dysregulation of the gut microbiota leads to an inability of the intestinal mucosal barrier to resist an infection with pathogenic bacteria and facilitates the occurrence of diseases affecting the normal physiological function of the host. Meanwhile, gut metabolites, such as short-chain fatty acids (SCFAs), can also modulate local or systemic immune responses (Schulthess et al., 2019). Studies have demonstrated that SCFAs can affect the differentiation of B cells and maintain the homeostasis of intestinal T cells (Scott et al., 2018; Rosser et al., 2020). Butyric acid, a representative SCFAs, is necessary for the colonization of intestinal bacteria (Barcenilla et al., 2000).

Recent studies have suggested that yeast β-glucan, a polysaccharide produced by fungi, can contribute to the differentiation of Th2 cells and maintain the steady-state of the intestinal immune system (Cao et al., 2018). Additionally, the gut microbiota maintains the immune homeostasis of the liver through the gut-liver axis (Heymann and Tacke, 2016). The liver proposed as an important innate immune organ contains a large number of immune cells, such as NK, Kupffer, and T cells. The activation of Kupffer and T cells can trigger the innate and adaptive immune response when the liver is continuously stimulated by antigens, pathogens, and endotoxins produced by the gut microbiota. This process can affect the tolerance of liver immunity (Milosevic et al., 2019; Albhaisi et al., 2020).

### Liver Disease

In recent years, the gut microbiota has received increasing attention because of its central role in host-microbiota interaction and its impact on liver diseases (Yang et al., 2020). A healthy gut microbiota can maintain liver metabolism and immune homeostasis. Meanwhile, this direct connection can cause several “negative effects.” Bacteria-derived products or toxins can get into the liver and disrupt its homeostasis if the liver immune system is pre-imbalanced or the natural barrier of the intestine is disrupted (Schnabl and Brenner, 2014). A large number of intestinal-derived toxic products enter the liver and are recognized by specific receptors, such as TLR. This can promote an inflammatory response, direct liver cell death, or chronic liver damage (Seki and Schnabl, 2012).

Bile acids and many other bioactive substances released by the liver enter the gut to be processed and metabolized by intestinal bacteria. On the other hand, the liver receives 70% of its blood supply from the gut via the portal vein, which contains metabolites, endotoxins, peptidoglycan and even microbiota. Thus, various types of liver cells, including hepatocytes, kupffer cells, hepatic stellate cells, sinusoidal cells, and biliary epithelial cells can respond to signals from the gut and affect liver function (Nicholson et al., 2012). The interaction between the gut microbiota and the liver involves multiple components, including metabolism, immunity, and neuroendocrine signals, and constitutes a complicated interaction network. The gut-liver axis plays an essential role in the pathogenesis of numerous liver diseases, such as alcoholic liver disease (ALD), chronic hepatitis, NAFLD, liver cancer, and drug-induced liver injury (Milosevic et al., 2019).

In general, increased intestinal permeability and bacterial translocation promote the arrival of microbial metabolites to the liver and aggravate the liver injury. This leads to dysfunctions in bile acid metabolism and intestinal peristalsis, systemic inflammation, and liver damage. The level of liver damage is closely related to the severity of intestinal disorders. Alteration of the bacteria that produce SCFAs, which dominate the composition of gut microbiota, has already been reported to affect intestinal homeostasis (Schulthess et al., 2019). Thus, a treatment based on microbial intervention is a promising method to improve the pathological process in the liver (Boursier et al., 2016).

At present, many studies have shown that supplementing with probiotics can balance the gut homoestasis, reduce intestinal bacterial translocation, inhibit liver inflammation, and improve acute liver damage Table 1 (Van Nood et al., 2013; Huang et al., 2018; Le Barz et al., 2019). Recent studies have also demonstrated that differences of distribution, specific composition and metabolites produced by the gut microbiota constitute the risk for acute liver injury, the major ways for the gut microbiota participate in ALI (Gong et al., 2018; Dey, 2020). Thus, the specific mechanisms of gut microbiota in the onset and progression of ALI deserve to further study. Considering the different types of ALI, we summarized the currently reported four chemical-induced acute liver injury model that modulated by gut microbiota.

**Table 1:** Application of probiotics in chemical-induced acute liver injury model.

**References**	**Probiotic**	**Animal**	**Chemical**	**Protective mechanism**
Jiang et al. (2021)	*Lactobacillus reuteri* DSM 17,938 (oral gavaged with 3 × 10^9^ CFU daily for 7 days)	Sprague-Dawley rats	D-GaIN, intraperitoneally injected of 1.1 g/kg body weight and sacrificed after injected 24 h.	Alleviate the disruption of the gut microbiota and metabolome; reduce the transcription of inflammatory factors in the liver
Saeedi et al. (2020)	*Lactobacillus rhamnosus* GG (oral gavage with 2x10^8^ CFU/100 μl HBSS daily for 14 days)	Germ-free C57BL/6 mice	APAP, oral gavage of 300 mg/kg bodyweight and mice were sacrificed after APAP gavage 24 h.	Production of 5-MIAA to activate Nrf2 in liver to protect against APAP induced oxidative liver injury
Neag et al. (2020)	MegaSporeBioticTM (MSB) (orally 1 × 10^9^ CFU/rat through a feeding tube daily for 12 days)	Charles River Wistar white male rats	APAP, oral gavage of 2 g/kg bodyweight and mice were sacrificed after APAP gavage 48 h.	Reduced the pro-inflammatory cytokines (TNF-α, IL-1β), decreased the hepatocyte necrosis
Li et al. (2019a)	*Bifidobacterium adolescents* CGMCC15058 (gavage 3 × 10^9^ CFU/ml PBS daily for 14 days)	Germ-free Sprague–Dawley (SD) rats	D-GaIN, intraperitoneally injected of 1.1 g/kg body weight and sacrificed after 24 h.	Decreased levels of mTOR and the inflammatory cytokines TNF-α and IL-6; increased the anti-inflammatory cytokine interleukin-10
Li et al. (2019b)	*Bacillus cereus* (gavage at 3 × 10^9^ CFU/ml PBS daily for 2 weeks)	Sprague-Dawley rats	D-GaIN, intraperitoneal injection of 1.1 g/kg body weight and sacrificed after 24 h	Alleviating the inflammatory reaction, reinforcing gut barrier function; reshaping the gut microbiota
Grander et al. (2018)	*Akkermansia muciniphila* (oral gavage1.5 × 10^9^ CFU/200 μl PBS daily for 2 days)	C57BL/6 mice	Alcohol, oral gavage of 6 g/kg bodyweight and mice were sacrificed after alcohol gavage 8 h	Reduced ethanol-induced hepatic injury, steatosis and infiltration of MPO^+^ neutrophils
Wu et al. (2017)	*Akkermansia muciniphila* (oral gavage 3 × 10^9^ CFU/200 μl PBS daily for 14 days)	C57BL/6 mice	Con A, 15 mg/kg injection through the tail vein and sacrificed after 8 h	Reduced inflammatory cytokines, cytotoxic factors and hepatocellular death; increased the diversity and reshaping the microbial community
Yu et al. (2017)	*Saccharomyces boulardii* (gavaged with 1 × 10^9^ CFU/ml for 4 weeks)	BALB/c mice	D-GaIN, intraperitoneally injected with 200 mg/kg body weight and sacrificed after 24 h.	Increased the relative abundance of *Bacteroidetes*; decreased the relative abundance of *Firmicutes* and *Proteobacteria*
Wang et al. (2016)	*Lactobacillus casei* Zhang (gavaged with 10^9^ CFU/daily for 30 days)	Wistar rats	LPS / D-GalN, intraperitoneal injection of 50 μg/kg LPS and 300 mg/kg D-GalN and sacrificed after 8 h.	Modulation of the TLR-MAPK-PPAR-γ pathways to reduce pro-inflammatory cytokines and hepatic inflammation
Wang et al. (2012)	*Lactobacillus rhamnosus* GG (mixed with drinking water approximately 10^9^ CFU /daily for 5 days)	C57BL/6N mice	Alcohol, oral gavage of 6 g/kg bodyweight and mice were sacrificed after alcohol gavage 1.5 or 6 h.	Activating HIF signaling to decrease the damage of alcohol-induced increased intestinal permeability and endotoxemia.

*D-GaIN, D-galactosamine; APAP, acetaminophen; 5-MIAA, 5-methoxyindoleacetic acid; Con A, Concanavalin A*.

## The Gut Microbiota and Apap-Induced Liver Injury

Drug-induced liver injury (DILI) is the main cause of clinical liver injury in developed countries. For example, in the United States, nearly 46% of ALI cases were caused by overdose or abuse of N-acetaminophen (APAP). APAP has been the focus of the Food and Drug Administration (FDA) advisory committees over the past several decades (Bernal et al., 2015; Lee, 2017). The degree of liver injury is the determining factor in acute liver failure survival without transplantation. During the past few decades, around three-quarters of APAP-induced ALI patients survive with their naïve liver (Stravitz and Lee, 2019). Generally, APAP is a safe drug when used at therapeutic doses for the treatment of fever and pain (1–4 g/days) (Kaplowitz, 2004). Its safety margin is, however, relatively narrow (Maeda et al., 2020). A randomized study reported that a maximum dose of APAP for 5 days in healthy adults increased the level of serum transaminase (Watkins et al., 2006). Meanwhile, the susceptibility to APAP-induced liver injury may also be due to several risk factors, such as alcohol use, obesity, nutritional depletion, and the use of drugs that stimulate the cytochrome P450 (CYP) system (Bunchorntavakul and Reddy, 2013).

Typically, APAP is cleared in the liver primarily where it binds to O-sulfuric acid or glucuronic acid and is excreted into the bile or urine. At therapeutic doses, only a small amount of APAP is metabolized by the cytochrome P450 enzyme (CYPs) to N-acetyl-p-phenyl quinone imine (NAPQI). With high doses of APAP overdose or if the ability of glucuronide and sulfate esterification is saturated (Mitchell et al., 1974), CYPs (mainly CYP-2E1 and CYP-3A4 in mammals) become the major APAP metabolic enzyme and lead to the massive production of NAPQI, a highly active intermediate compound that leads to cytotoxicity (Lee et al., 1996; Laine et al., 2009). In the early stage, the majority of NAPQI is rapidly detoxified and excreted into the urine by binding with glutathione (GSH). Once the GSH storage is depleted, the remaining NAPQIs accumulate in hepatocytes and bind covalently to proteins sulfhydryl groups, producing harmful APAP protein adduct (APAP-ADs) that irreparably lead to liver cell necrosis (Jaeschke et al., 2012; Mcgill and Jaeschke, 2013).

N-acetylcysteine (NAC), the only approved therapeutic drug for APAP-induced hepatotoxicity, can protect the liver from damage by providing cysteine precursors and restore the hepatocytes GSH stores (Ferner et al., 2011). However, the treatment window for NAC is narrow and requires a controlled use of APAP (no more than 24 h). The accumulation of NAPQIs can destroy cytoplasmic membranes and induce severe mitochondrial dysfunctions, an overproduction of reactive oxygen species (ROS), ATP depletion, a fragmentation of nuclear DNA, and lipid peroxidation (Jaeschke et al., 2012; Ramachandran and Jaeschke, 2020). Therefore, massive hepatocellular necrosis subsequently leads to several damage-associated molecular patterns (DAMPs) releases, which activate macrophages and induce a sterile inflammation (Jaeschke and Ramachandran, 2020). Activated macrophages produce several chemokines and cytokines, which induce the intrahepatic aggregation of neutrophils, monocytes, and other immune cells and trigger intrahepatic and systemic inflammatory responses (Yang and Tonnesseen, 2019). Notably, sterile inflammatory responses not only clear necrotic cell debris and promote tissue repair, but also aggravate liver injury (Jaeschke and Ramachandran, 2020). Finally, when the degree of hepatocyte damage exceeds regeneration, the liver function rapidly fails.

The hepatotoxic effect of paracetamol is still considered to be the most important part of ALI. However, recent evidence suggests that changes in the gut microbiota, including their abundance, diversity, metabolites, intestinal permeability, and bacterial translocation also play profound roles in APAP-induced hepatotoxicity (Dey, 2020).

Several studies have shown that the presence of gut microbiota is important for APAP-induced hepatotoxicity. Jourova et al. found that the degree of APAP liver injury in germ-free (GF) mice or mice cleared of gut microbiota was lower than in SPF mice. The expression of the P450 enzyme, which is involved in APAP metabolism in the liver, is closely linked to intestinal microorganisms (Jourova et al., 2020). The expression levels of CYP-1A2 and CYP-3A4 in the liver of GF mice were significantly lower than in SPF mice, which may explain the significant reduction of APAP-induced hepatotoxicity in GF mice (Jourova et al., 2020). Meanwhile, single-cell sequencing data showed that there were significantly fewer *LY6C*-positive monocytes and a lower proportion of activated non-parenchymal cells (hepatic stellate cells, sinusoidal endothelial cells, and Kupffer cells) in the liver of antibiotic-treated mice compared with SPF mice. The key mechanism depends on the clearance of intestinal microbiota, which dramatically reduces the microbial-associated molecular patterns (MAMPs) entering the liver via the portal vein. Blocking the TLRs-MYC signaling pathway of MAMPs can downregulate the activation of stellate, endothelial, and Kupffer cells and significantly decrease the expression of chemokines and pro-inflammatory mediators, thus reducing the intrahepatic inflammatory response in APAP-induced model (Kolodziejczyk et al., 2020). These results suggested that single cell sequencing is an important method to study the direct relationship between gut microbiota and liver.

Similarly, the change in diversity and abundance of the intestinal microbiota can also participate in the transformation and detoxification of APAP. Zheng et al. changed the composition of the gut microbiota in mice using vancomycin, which reduced the abundance of Gram-positive bacteria in the intestine and increased the level of 2-hydroxybutyric acid in the cecum and serum. The bioavailability of APAP was also decreased and the level of GSH in the liver was increased, which improved the APAP-induced liver injury in mice (Zheng et al., 2020). The pharmacokinetics of APAP is also affected by changing the composition of gut bacteria in mice. Several researchers showed that the degradation of APAP increased by 68% in mice treated with *Lactobacillus reuteri*, while treatment with *Lactobacillus rhamnosus* did not show a similar effect (Kim et al., 2018).

The susceptibility to APAP toxicity varies considerably among different individuals. Endogenous p-cresol, a protein residue in the intestinal cavity, is mainly produced by intestinal microorganisms, such as *Clostridium difficile*. P-cresol absorbed into the liver is transformed into p-cresol sulfate by sulfotransferases in hepatocytes (Bone et al., 1976). Likewise, acetaminophen is a substrate of sulfotransferases and p-cresol can reduce the ability of sulfotransferases to sulfonate acetaminophen. The content of p-cresol varies greatly in different individuals, which may explain the different sensitivity of individuals to APAP hepatotoxicity (Clayton et al., 2009). Furthermore, individuals with intestinal dysbiosis are more sensitive to APAP-induced liver injury. Schneider KM et al. analyzed a cohort of 500,000 participants in the British Biobank and found that proton pump inhibitors (PPI) or long-term antibiotics (ABX) can cause intestinal microbial dysbiosis. The risk of ALF induced by APAP was significantly increased in participants with intestinal microbial dysbiosis (Schneider et al., 2020). Similarly, compared with wild-type mice, *Nlrp6*^−^^/−^ mice (an intestinal dysbiosis model) also showed that microbial dysbiosis could aggravate APAP-induced liver injury. This phenotype was reproduced after fecal bacteria transplantation (Elinav et al., 2018; Schneider et al., 2020).

Microbial communities are altered by the circadian rhythm system and affect the host metabolism, energy homeostasis, and immune system (Teichman et al., 2020). Disruptions in the rhythm function of microbe-host interactions can seriously affect the pathology and severity of the disease (Bishehsari et al., 2020). Interestingly, changes in the gut microbial circadian rhythm can affect APAP-induced liver toxicity (Gong et al., 2018). 16S rRNA gene sequencing showed that changes in the daily rhythm were associated with changes in the relative abundance of gut microbiota. Compared with ZT0, the ratio of Firmicutes/Bacteroides in ZT12 was significantly reduced. The hepatotoxicity of APAP was more severe at night (ZT12) than in the morning (ZT0). This was likely due to the increased abundance of the metabolite 1-phenyl-1,2-propanedione (PPD), which can consume GSH in hepatocytes after being ingested by the liver through the portal vein at ZT12. Therefore, early consumption of GSH can cause the accumulation of a large amount of NAPQI in the liver of mice with an excessive APAP metabolism, which can aggravate the liver injury. The oral gavage of APAP weakens the intestinal mucosal barrier function during intestinal absorption and allows plasma albumin to seep into the intestinal cavity. Similarly, a higher intestinal permeability can also allow abundant harmful substances to enter the liver, which aggravates the inflammatory reaction and the hepatotoxicity of APAP (Schneider et al., 2020). Niu et al. confirmed that the disruption of the intestinal barrier integrity, which may be mediated by intestinal immune microenvironments, can aggravate APAP-induced hepatotoxicity (Niu et al., 2020).

A high concentration of APAP can rapidly induce the apoptosis of *Lgr5* ^+^ crypt basal stem cells in the small intestine. Although apoptotic cells are completely removed within 24 h, the potential consequence is a long-term defect in the intestinal barrier function (Chopyk et al., 2019). Apoptosis of *Lgr5*^+^ crypt basal stem cells may partly explain why the low 30-days survival rate of ALF caused by APAP toxicity in liver transplant recipients compared with ALF caused by non-APAP-related causes (Cooper et al., 2009).

Probiotics restore the balance of the intestinal microbiota (symbiotic and pathogenic bacteria), maintain the integrity of the intestinal barrier, reduce the production of toxic products, and improve liver function. We wondered, however, how probiotics could affect the APAP-induced hepatotoxicity. It is a known fact that gut microbiota and its metabolites are involved in the regulation of oxidative stress and inflammation which play key roles in drug-induced hepatotoxicity (Jaeschke et al., 2004; Shehu et al., 2017). Many studies showed that, compared with GF mice, normal mice displayed an upregulation of the transcription factor Nrf2 in liver that improved the antioxidant and xenobiotic response to protect the liver from acute acetaminophen; this signaling upregulation is enhanced by supplementary of human commensal *Lactobacillus rhamnosus GG* (Saeedi et al., 2020). Meanwhile, Sharma et al. reported that probiotic *Enterococcus lactis IITRHR1* and *Lactobacillus acidophilus MTCC447* protect against APAP-induced liver injury by modulating the antioxidant capacity of the liver and the expression of key apoptotic/anti-apoptotic proteins (Sharma et al., 2011). MegaSporeBioticTM probiotic capsules are composed of a probiotic blend of spores from five Bacillus species that improve the histopathological hepatic injury and decreased the level of proinflammatory cytokines, indicating that *Bacillus spa* spores have a protective effect on acute hepatic injury induced by APAP (Neag et al., 2020). This data is significant for the treatment of ALI and we also need to further explore whether the pretreatment of probiotics could improve the detoxification ability and antioxidant capacity of hepatocytes.

## The Gut Microbiota and Con A-Induced Autoimmune Hepatitis

Autoimmune hepatitis (AIH) is a complicated immune-mediated liver disease with a variable clinical phenotype that occurs worldwide. In the UK, the incidence rate of AIH reaches nearly 1.94 cases per 100,000 people (Webb et al., 2021). The histological diagnosis of AIH comprises interfacial hepatitis, increased serum alanine aminotransferase (ALT), increased aspartate aminotransferase (AST), elevated immunoglobulin G (IgG) levels, and the presence of autoantibodies (Floreani et al., 2018). Additionally, patients with acute severe autoimmune liver disease are more likely to develop acute liver failure and may need LT. However, autoimmune liver disease can still develop or recur in allografts with a 5-years recurrence rate of 36% to 68% after LT (Kerkar and Yanni, 2016). The mechanism of AIH recurrence after LT is still unclear. However, several extrahepatic factors may play an important role in the recurrence of AIH. Mounting evidence showed that the mechanisms of AIH were related to gut dysbiosis (Cai et al., 2017; Furukawa et al., 2020). Moreover, microbiome restoration therapies, such as probiotics, prebiotics, and fecal microbiota transplantation (FMT), can effectively improve AIH (Allegretti et al., 2019; Zhang et al., 2020). The study of gut microbiota will provide new insights into the mechanism of AIH.

Due to the lack of widely accepted and valid mouse models for AIH, the research on the pathogenesis of AIH is still very limited. Concanavalin A (Con A) is a lectin originally extracted from jack-bean. In early 1992, it was first used to establish an immune hepatitis model (Tiegs et al., 1992). Con A-induced hepatitis is a typical chemical model that is used to investigate the cellular and molecular mechanism of immune-mediated liver injury (Tiegs et al., 1992; Fujita et al., 2016; Xu et al., 2018). The model is acute and the injury caused by Con A usually lasts for only 48 h. Con A can partly simulate the pathogenesis of human acute autoimmune liver diseases as it can rapidly induce the activation of natural killer T (NKT) cells and CD4 positive T cells in the mice liver and release a mass of cytokines to cause liver damage (Diao et al., 2004; Celaj et al., 2014). Here, we mainly summarize the role of gut microbiota in Con A-induced hepatitis and summarize the discovery and progress of immunotherapy related to the gut-liver axis.

NKT cell activation plays a critical role in Con A-induced hepatitis and the gut microbiota regulates the activation of NKT cells (Diao et al., 2004; Celaj et al., 2014). Wei et al. have shown that GF mice are not sensitive to Con A-induced liver injury when compared with SPF mice. This is mainly because NKT cells in the liver of GF mice are not activated after Con A treatment. Meanwhile, compared with GF mice, Con A treatment can significantly increase the circulation of LPS and the level of glycolipid antigen presented by CD1d in SPF mice. Intestinal microbial-derived antigens (glycolipids) are also important activators of liver NKT cells as they can activate them to mediate Con A-induced ALF (Wei et al., 2016).

Furthermore, Chen et al. showed that exogenous pathogenic bacteria exposed to the gut can exacerbate Con A-induced liver injury. This may be due to the increase of DCs activation, which subsequently augments the cytotoxicity of hepatic NKT cells against the liver parenchyma cells. In contrast, gentamicin treatment, which is bactericidal mainly against gram-negative (G^−^) organisms in the gut, can alleviate Con A-induced ALI (Chen et al., 2014). Meanwhile, Celaj et al. demonstrated that differences in the gut microbiota could determine the sensitivity to Con A-induced acute liver injury. They found that mice from the Taconic Farms (TAC), and the Jackson Laboratory (JAX) exhibited different levels of liver damage induced by Con A. This difference in sensitivity is caused by the regulation of the Fas response pathway in the gut microbiota. Interestingly, this difference in susceptibility disappeared after co-housing (a way for minimizing the discrepancy of gut microbiota from the different background). By analyzing the fecal microbe by 16S rRNA gene sequencing, they found that among the identified OTUs, 8 genera abundance exhibited statistically significant differences in the two company's mice. Furthermore, they found the abundance of Ruminococcaceae was positively associated with the degree of liver injury (Celaj et al., 2014). Dopamine is an important neurotransmitter and also an immune modulator. It participates in the regulation of the T cell function through the D1-like receptor (Besser et al., 2005; Nakano et al., 2011). Xue et al. demonstrated that gut microbes can improve the synthesis of peripheral dopamine to inhibit the activation of hepatic iNKT cells and alleviate Con A-induced liver injury. Different with Wei et al. study in GF mice, the depletion of the gut microbiota by antibiotics reduced the synthesis of dopamine and exacerbated Con A-induced liver injury (Xue et al., 2018). These conflict phenotypes may be resulted from the difference of microbiota abundance between GF and antibiotics treated mice. Nobuhito et al. showed that inducing colitis with DSS for 7 days led to intestinal leakage, exposed intestinal microbes to the systemic immune cells and the liver through the portal vein, induced a systemic immune tolerance, and thereby reduced Con A-induced liver damage (Taniki et al., 2018).

Due to the high plasticity of the intestinal microbiota, the research of exogenous probiotics or prebiotics in autoimmune liver disease is progressing substantially. However, there are few reports about the impact of probiotics or prebiotics on Con A-induced immune liver injury. The gut microbiota may influence the susceptibility and severity of acute liver injuries caused by Con A in mice based on current evidence. Gabriela et al. reported that *Propionibacterium acidipropionici* CRL 1,198 decreased the proliferative effects of lectins in adenocarcinoma cells, inhibited the fermentative activity of colonic microbiota, and avoided undesirable lectin-epithelia-microbiota interactions (Zarate et al., 2017). As a promising probiotic with beneficial effects on liver diseases, *Akkermansia muciniphila* pre-treatment can also alleviate liver damage by altering transaminase activities and attenuate systemic inflammation by suppressing cytokines (including IFN-γ, IL-1β, IL-2, and IL-12p40) in Con A-induced liver injury. The beneficial effects of *A. muciniphila* were partly dependent on improved intestinal barrier and restored composition and function of gut microbiota (Wu et al., 2017).

Therefore, the study of the relationship between intestinal microorganisms and liver immune homeostasis is helpful for the diagnosis and treatment of acute autoimmune liver injuries.

## The Gut Microbiota and Alcohol-Induced Acute Liver Injury

Alcohol (ethanol) abuse is a common risk factor for multiple diseases including alcoholic liver diseases (ALD), cardiovascular diseases, and inflammatory bowel diseases (Connor et al., 2016). Every year, nearly 4% of adults get sick because of drinking and the harmfulness of alcohol even exceeds the perniciousness of smoking (Room et al., 2005). Alcohol is transformed by alcohol dehydrogenases and cytochrome CYP-2E1 to acetaldehyde in the endoplasmic reticulum of hepatocytes. Acetaldehyde is converted into acetic acid by acetaldehyde dehydrogenases and is finally excreted in urine (Arteel, 2003). However, the gut microbiota also have a crucial part to play in the metabolism of ethanol because a large number of bacteria can express alcohol dehydrogenases in the colon. Evidence showed that a leaky gut, bacterial translocation, and intestinal inflammation modulate the susceptibility of acute ALD (Starkel et al., 2018).

Canesso et al. showed that compared with GF mice, administration of alcohol for 7 days can cause significant liver injury and increase the level of neutrophil infiltration and pro-inflammatory cytokines (CXCL-1 and interleukin (IL)-6) in SPF mice. This implied that the gut microbiota was directly or indirectly involved in acute ALD (Canesso et al., 2014). However, our data showed that a single gavage of a high concentration of alcohol (300 μl of 30% (vol/vol) EtOH at a dose of 3 g/kg) caused more severe liver damage, inflammation, hepatic steatosis, and higher levels of CYP-2E1 in GF mice than in SPF mice, which also indicated that the intestinal microbiota was directly or indirectly involved in alcohol-induced liver damage (Chen et al., 2015). According to these results, the time and concentration of alcohol exposure may explain the inconsistencies between the two programs.

To date, several studies showed that changes in the entero-hepatic axis, such as increased permeability of the intestinal barrier, a thinning of the protective mucosal layer, and changes in the gut microbiota, occurred after alcohol consumption (Carson and Pruett, 1996; Purohit and Brenner, 2006). *Akkermansia muciniphila*, a Gram-negative bacteria, can enhance mucus production to improve intestinal barrier function. Studies have documented that *A. muciniphila* abundance is significantly decreased in patients with alcoholic hepatitis and mice exposed to alcohol. Interestingly, the presence of alcohol did not affect the commensal *A. muciniphila* growth *in vitro* (Grander et al., 2018). The mechanism by which ethanol depletes *A. muciniphila* remains unclear. Lee et al. demonstrated that a low-dose (0.8 g/kg/days) of alcohol for 7 days can change the fecal microbiota composition, while fermented rice liquor can restore the microbial composition and inhibit intestinal inflammation (Lee et al., 2020).

The regulation of the gut microbiota appears to be a promising strategy to improve acute ALD (Vassallo et al., 2015). Pharmacological research showed that several drugs could regulate the composition of the gut microbiota and treat acute ALD. Audrey et al. proved that rhubarb extracts increased the abundance of mucus *A. muciniphila* and *Parabacteroides goldsteinii* by decreasing the activation of the TLR4 pathway and reducing the levels of inflammation and oxidative stress in the liver tissue, which improved acute liver injury caused by alcohol (30% w/v, 6 g/kg body weight) (Uesugi et al., 2001; Neyrinck et al., 2017). Meanwhile, there have been reports on the intervention of the gut microbiota in acute alcoholic liver disease. The supplementation of *Pediococcus pentosaceus* CGMCC 7,049 improved the intestinal barrier function and reversed gut microbiota dysbiosis by reducing the level of circulating endotoxin and proinflammatory cytokines (Jiang et al., 2020).

Animal studies have shown that probiotics and synbiotics improved gut microbiota disorders, enhanced the mucosal barrier, and protected hepatocyte from acute and chronic ethanol injury (Han et al., 2020; Lee et al., 2020). However, clinical studies data is still insufficient and we need further investigation in the future.

## The Gut Microbiota and D-Galactosamine-Induced Acute Liver Injury

D-galactosamine (D-GalN), a hexosamine derived from galactose, is a component of various glycoprotein hormones and a hepatotoxic agent. Intraperitoneal injection of D-GalN can cause diffuse hepatocyte necrosis and inflammation analogous to the changes of the liver pathology after clinical viral hepatitis (Keppler et al., 1968; Rahman et al., 2018). In general, the co-administration of a sublethal dose D-GalN and lipopolysaccharide (LPS) has been widely used in repetitive experimental animal models of fulminant liver failure in the clinic. The hepatotoxicity induced by D-GalN is mainly due to a decrease in the concentration of uridine diphosphate in hepatocytes and the methylation of ribosomal RNA, which can affect the normal translation of proteins. This results in the inhibition of RNA and protein synthesis and the disruption of the normal hepatocytes metabolism, which leads to cell necrosis and inflammatory infiltration (Clawson et al., 1990; Kmiec et al., 2000). It has been reported that D-GalN also affects the synthesis of cell membrane components and eventually causes hepatocyte death (Decker and Keppler, 1974).

We recently found LPS/D-GalN administration could rapidly change gut microbial function which may further influence intestinal soyasaponin II level. Soyasaponin II exerts anti-inflammatory effect by targeting Y-Box Binding Protein 1 and Nlrp3 inflammasome (Wang et al., 2020a). Beside our work, accumulating evidence suggests that D-GalN treatment not just induces hepatocyte damage but also destroys the gut microbiota homeostasis and the intestinal barrier structure by allowing MAMPs, such as LPS, to enter the liver and peripheral blood (Li et al., 2004). Conversely, pretreatment with probiotic *Lactobacillus reuteri* DSM 17938 can relieve the gut dysbiosis, reduce the transcription of inflammatory factors, and alleviate D-GalN-induced liver injury in rats (Jiang et al., 2021). Probiotic *Lactobacillus casei Zhang* can also reduce LPS/D-GalN-induced pro-inflammatory cytokines and hepatic inflammation through the modulation of the TLR-MAPK-PPAR-γ signaling pathways (Wang et al., 2016). Li et al. data showed that the oral gavage of *Bacillus cereus* for 15 days before D-GalN administration significantly improved liver injury and inflammatory processes by decreasing plasma endotoxin levels, reinforcing the gut barrier function, and improving the gut microbiota (Li et al., 2019b). Wang et al. demonstrated that Sprague-Dawley rats pretreated with *Lactobacillus helveticus* R0052 for 7 days showed a significant reduction in the levels of ALT, bilirubin, and total bile acid that were changed by D-GalN. Additionally, R0052 exhibits anti-inflammatory properties by down-regulating the transcription of Toll-like receptors, tumor necrosis factor-α, and nuclear factor-kappa beta (NFκβ) in liver tissues. R0052 can also improve intestinal lactic acid bacteria and Bacteroides (Wang et al., 2019). Similarly, *Bifidobacterium adolescents* CGMCC15058, *Bifidobacterium longum* R0175, and *Saccharomyces boulardii* reduce the increase of cytotoxic factors and inflammatory cytokines in D-GalN-induced liver injury in rats (Yu et al., 2017; Li et al., 2019a; Wang et al., 2020b). In brief, D-GalN is a widely used model to examine the protective mechanism of exogenous probiotics, prebiotics on fulminant liver failure.

## Conclusion

**Figure 2:**
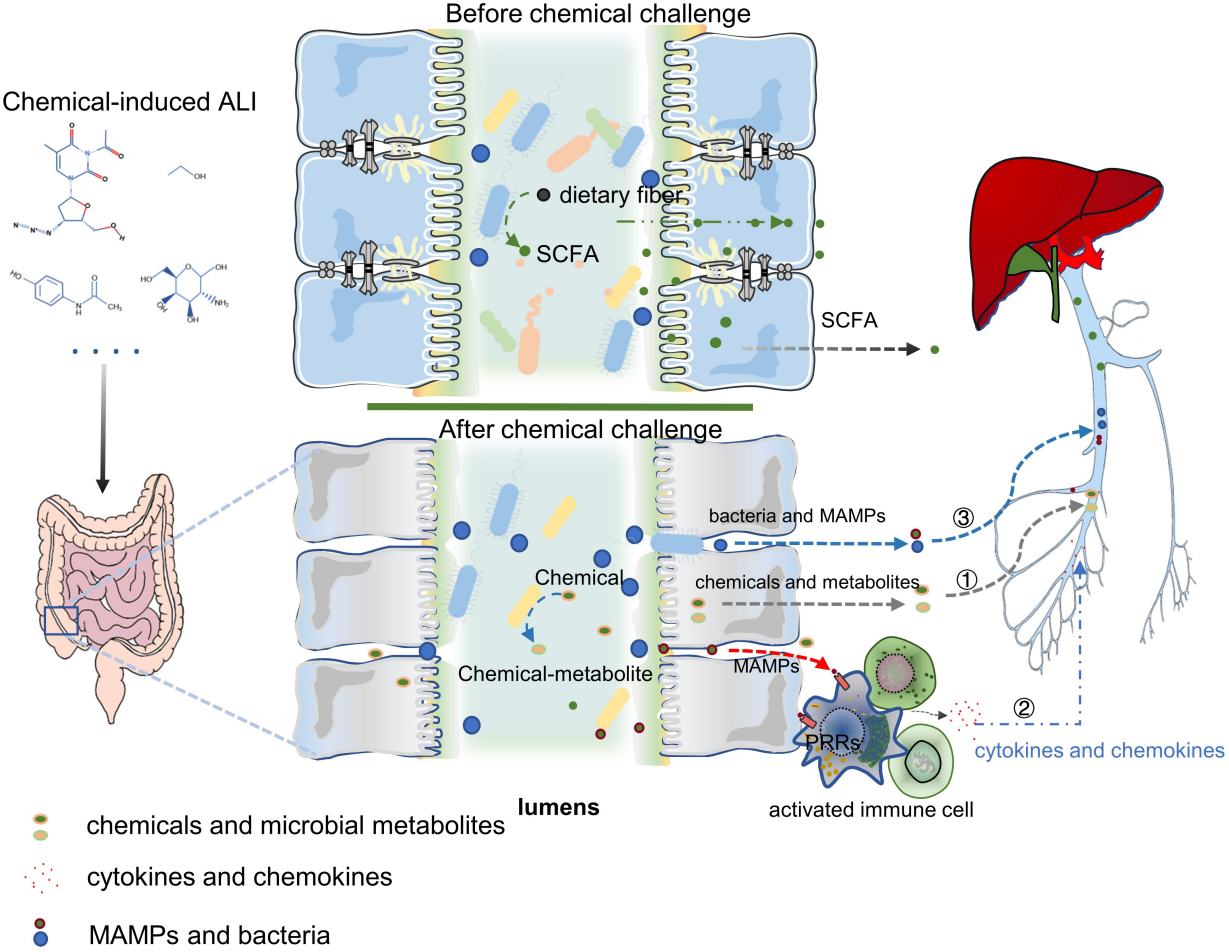
The mechanism of gut microbiota in ALI can be summarized by three points: ① Chemicals transformation and metabolites of gut microbiota can influence the hepatocyte's ability to metabolize toxic substances and affect hepatic function; ② Gut harmful bacteria and MAMPs activate the systemic immune response, release a large number of chemokines and pro-inflammatory cytokines into the liver, leading to liver inflammation; ③ Intestinal mucosal barrier destroy (containing the weakening of the mucus barrier, the destruction of cell tight junction, and the necrosis of intestinal epithelial cells) increases bacterial translocation and the entry of MAMPs into the liver, which mediates liver damage. PRRs, Pattern Recognition Receptors. Figure was partly adapted from Smart Servier Medical Art (https://smart.servier.com/), which is licensed under a Creative Common Attribution 3.0 Generic License.

Acute liver injury is a life-threatening disease with various causes and rapid progress. The application of chemical-induced liver injury models is of great significance for the study of the pathophysiological mechanism of ALI. In recent years, with the development of rapid, sensitive, and cheap gene sequencing technology and omics technology, the gut microbiota was being found to play a key role in host liver immunity, metabolism, and detoxification. The role of gut microbiota in ALI has also been gradually revealed. In the chemical-induced ALI models, the main mechanisms of gut microbiota in regulating ALI can be summarized by three points (Figure 2). Firstly, the intestinal microbes directly affect the detoxification ability of hepatocyte through the changes of bacterial metabolites and the transformation of chemical toxins; secondly, microbes and MAMPs are exposed to the systemic immune cells through the damaged intestinal mucosal barrier. Then the immune cells were activated, which release a large number of chemokines and pro-inflammatory cytokines into the liver, and induce liver inflammation. Finally, gut microbiota dysbiosis or chemical poisons directly induced the intestinal mucosal barrier damage (including weakened mucus barrier, destructed cellular tight junctions and the necrosis of intestinal epithelial cells), increase bacteria and MAMPs translocation into the liver, and mediate liver injury. In future, using single-cell RNA sequencing might help to further study how the gut microbiota directly affects hepatocytes and liver non-parenchymal cells. Numerous studies have shown that exogenous supplementation with prebiotics and probiotics can improve various chemical-induced ALI models. These data suggest that modulation of the gut microbiota for applying to clinical treatment of ALI is one possibility. The gut microbiota is a multifaceted community but nearly all of the research have focused on the role of bacterial disturbance in ALI models. The progress of fungi and viruses in this field is very limited. Future studies are required to better understand the systemic role of the gut microbiota; including viruses, fungi, and parasites, in the occurrence and development of ALI.

## Author Contributions

TC and RL draft the manuscript. PC edit the manuscript and supervised the work. All authors contributed to the article and approved the submitted version.

## Conflict of Interest

The authors declare that the research was conducted in the absence of any commercial or financial relationships that could be construed as a potential conflict of interest.
